# Investigation of fullerenol-induced changes in poroelasticity of human hepatocellular carcinoma by AFM-based creep tests

**DOI:** 10.1007/s10237-017-0984-5

**Published:** 2017-12-01

**Authors:** Xinyao Zhu, Srdjan Cirovic, Aliah Shaheen, Wei Xu

**Affiliations:** Faculty of Engineering and Physical Sciences, University of Surrey, Guilford, GU2 7XH UK

**Keywords:** Atomic force microscope, Poroelastic, Permeability, Diffusion, Fullerenol, Creep

## Abstract

In this study, atomic force microscopy (AFM) is used to investigate the alterations of the poroelastic properties of hepatocellular carcinoma (SMMC-7721) cells treated with fullerenol. The SMMC-7721 cells were subject to AFM-based creep tests, and a corresponding poroelastic indentation model was used to determine the poroelastic parameters by curve fitting. Comparative analyses indicated that the both permeability and diffusion of fullerenol-treated cells increased significantly while their elastic modulus decreased by a small amount. From the change in the trend of the determined parameter, we verified the corresponding alternations of cytoskeleton (mainly filaments actin), which was reported by the previous study using confocal imaging method. Our investigation on SMMC-7721 cell reveals that the poroelastic properties could provide a better understanding how the cancer cells are affected by fullerenol or potentially other drugs which could find possible applications in drug efficacy test, cancer diagnosis and secure therapies.

## Introduction

Since the discovery of buckminsterfullerene ($$C_{60})$$ in 1985, fullerenes have drawn attention due to its potential function in biomedical areas, e.g., cancer diagnosis and therapy (John son-Lyles et al. 2010; Chen et al. 2012). Despite of its promising medical prospects, $$C_{60}$$ has inferior solubility in aqueous solutions which prevents its popularity in biological applications (Partha and Conyers 2009). However, this issue is circumvented to a great extent by chemical or supramolecular method, and thus various functionalized fullerenes have been compounded to achieve promising results (Bosi et al. 2003). Fullerenol, being a fullerene derivative by adding hydroxyl groups onto fullerene molecules, has been documented in the literature to possess a potential in antioxidant, antitumor and antimetastatic activities (Rade et al. 2008; Bosi et al. 2003; Chen et al. 2012; Lu et al. 1998). They are associated with suppress of the synthesis of microtubules and disruption of actin filaments (Mrdanović et al. 2009; Johnson-Lyles et al. 2010), which not only act as a framework of the cytoskeleton but also regulate mechanical stability of living cells (Hawkins et al. 2010; Unterberger et al. 2013).

The dynamic alternations in a cytoskeleton are correlated with cell behaviors, e.g., cell growth, differentiation, apoptosis, proliferation and metastasis. Alternations of cyto-mechanical properties present a straightforward reflection of changes in cytoskeleton and hence cellular physiological and pathological processes (Aryaei and Jayasuriya 2013; Etienne-Manneville 2004; Nikolaev et al. 2014). It is known that the cytoplasm is the largest part of cell by volume and thus its rheology affects the rate at which the profile of cell alters. Some recent experimental results suggested that the rheology of cytoplasm could be represented using a poroelastic model, where a porous elastic solid meshwork (e.g., cytoskeleton, organelles and macromolecules) is steeped in an interstitial fluid (cytosol) (Moeendarbary et al. 2013; Leipzig and Athanasiou 2005). In this model, the ability of liquid to flow through cell’s meshwork, i.e., the permeability, is critical to the mechanical behavior of the living cell (Berteau et al. 2016). On the other hand, the elasticity of solid meshwork is characterized by the shear modulus *G* which reflects the cell’s response to shear stress and is independent of flow (Selvadurai 2004).

Previous studies have measured changes of elastic properties in cancerization or cancerous cell treatment with certain drugs, but the changes in the poroelastic properties due to drug treatment is yet to be explored in depth. Since cytoskeleton disruption is associated with drug treatment, the poroelastic properties of cancer cells would be affected by the fullerenol treatment. In this regard, the investigation of the poroelastic properties of cancer cells treated with fullerenol could provide valuable biomechanical information about how this drug affects the biphasic components of cancer cells, which will enhance the comprehension of the role and influence of fullerenol acting as an anticancer agent.

In this study, human hepatocellular carcinoma (SMMC-7721) cells, being one of the most common types of cancers worldwide, are used as samples. Both control and fullerenol-treated cancer cells are subjected to creep tests achieved using AFM indentation. By fitting the force-creep curves with the poroelastic model, three key parameters were extracted, i.e., elastic modulus, Poisson’s ratio and intrinsic permeability, we found that the treatment of fullerenol exhibits a sharp increase in permeability and diffusion (derived from the above parameters) while a small decrease in elastic modulus. The trend of these changes reflect the corresponding alternations in cytoskeleton ascribed to fullerenol treatment, which is also revealed by confocal imaging by the other studies. Moreover, we also compare the differences between the elastic modulus determined by Hertz model using different loading rates. To the best of our knowledge, this study presents a first attempt to investigate the poroelastic properties of cancer cells and it is also a pioneer study using creep model to measure cyto-poroelastic properties. The objective of this study is to present a case how the quantification of poroelastic properties of single liver cancer cell treated with fullerenol could be used to evaluate the effect of fullerenol or other anticancer agents on the cells. The potential significance of this study lies that it nominates poroelastic properties as a biomarker for drug efficacy testing and even cancer diagnosis.

## Materials and methods

### Cell preparation

The liver cancer (SMMC-7721) cells were purchased from the Cell Bank of the Shanghai Institute of Cell Biology, Chinese Academy of Sciences. The cells were cultured in Roswell Park Memorial Institute (RPMI)-1640 medium containing 10% fetal bovine serum (FBS), 100 U/ml penicillin and $100~{\mu }\hbox {g/ml}$ streptomycin, incubated in a humidified atmosphere of 5% $\hbox {CO}_{2}$ and at a temperature of $37\,{^{\circ }}\hbox {C}$. After an exponential phase of 24-hour incubation, the cells adhered to the bottom of flask, and they were treated by trypsin so that they could detach the flask bottom. The detached cells were seeded in a 3.5 mm petri dish and incubated for another 24 h at $37\,{^{\circ }}\hbox {C}$ for fullerenol treatment. The commercial aqueous solution of fullerenol has a concentration of 2 mg/ml, and it was then diluted with RPMI-1640 media with 10% of fetal bovine serum to $0.53\,{\mu }\hbox {M/ml}$. Afterward, 2 ml of the diluted fullerenol solution was added to the aforementioned petri dish which contained the cells. For comparison, 2 ml of RPMI-1640 media with 10% of fetal bovine serum was added to the control cells.

### Creep tests using AFM

The AFM employed for this study is JPK NanoWizards 3 BioScience (Berlin, Germany), and it is mounted on an inverted optical microscope (Olympus IX71; Tokoy, Japan), allowing the AFM and optical microscope imaging simultaneously. The criterion for the cantilever selection is that the stiffness of it should be around the range of the compliance of cell sample. It is recommended that for the measurement on a soft and delicate cell, the spring constant of the cantilever should range from $0.01\, \hbox {to}\, 0.06~\,{N/m}$ (Neumann 2008). Before indentation, the spring constant of the AFM cantilever was calibrated. A silicon nitride cantilever (Novascan, technologies Inc., Ames, IA, USA) with the spring constant $0.059~\hbox {N/m}$ was used for the cell-tip indentation. The probe is a sphere made by polystyrene, and its radius is $1~{\mu }\hbox {m}$. A liquid cell for the AFM cantilever was used to perform the experiments in phosphate buffer solution. A closed-loop system was used to detect the position of the probe in *z* (vertical) direction during the creep test. The operation of AFM and optical microscope was controlled by JPK’s CellHesion${\circledR }$ 200 software. The whole system was mounted on an anti-vibration table (TMC 63-530, USA). The room temperature was $27\,{^{\circ }}\hbox {C}$ and variation of room temperature was less than $+/-1.0\,{^{\circ }}\hbox {C}$ during experiments.

### Loading details

**Fig. 1:**
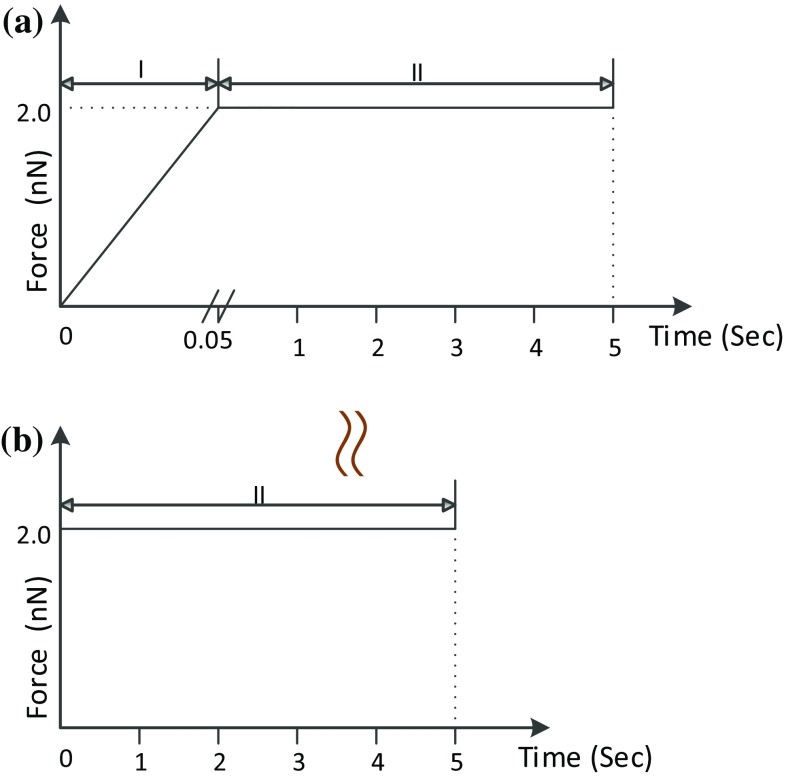
Schematic of the AFM indentation force versus time **a** and its approximation **b** by Heaviside step function.

In order to measure rate-dependent properties of material of interest, a common method is to monitor the creep response of the material subjected to an invariant force. To carry out the creep test on single cells, the loading history of indentation force is set as shown in Fig. 1a. The loading could be approximated by an Heaviside step function as shown in Fig. 1b, provided the ramping period (stage I) is much smaller than the characteristic time of the poroelastic materials (Chen et al. 2012), which will be discussed in Sect 3.3. In the present AFM indentation measurement, the constant force delay mode of the AFM was used to achieve the creep test. The force ramps to its maximum value (2*nN*) within 0.05 seconds and dwells at the peak value for 5 seconds, as shown in Fig. 1a.

Although viscoelastic models have been previously used to describe time-dependent behavior of cell mechanics, the recent studies indicate that the poroelastic model captures the time-dependent mechanics of cells at a short-time scale, i.e., less than 0.5 s (Chen 2014). Therefore, the poroelastic model appears to work better when a short ramping time involved.

**Fig. 2:**
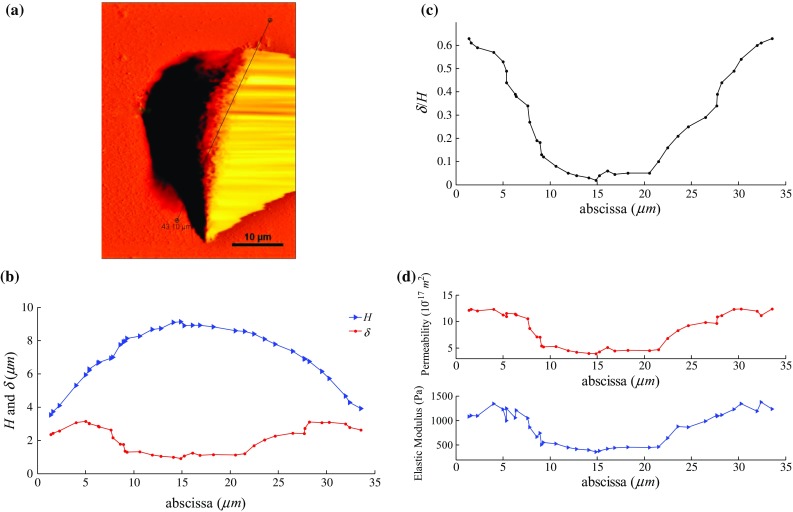
**a** The AFM topography image of living liver cancer cell in culture medium at $27\,{^{\circ }}\hbox {C}$. **b** Cell height profile, indentation depth **c** $\delta$/*H* and **d** poroelastic parameters variation along cut path

### Theoretical model

The full fundamental knowledge of poroelasticity and its application in nanoindentation used in our study is well documented by Wang (2000) and Selvadurai (2004), respectively. The sample cell in the present study is considered as a linear isotropic poroelastic material. This kind of constitutive model is dominated by five parameters: shear modulus *G*, the undrained Poisson’s ratio ${\nu }_{u}$, drained Poisson’s ratio $\nu$, the hydraulic (Darcy) permeability $\kappa$ and the effective stress coefficient $\alpha$ by which pore pressure reduces the matrix stress. The value of the undrained Poisson’s ratio is fixed at 0.5, and the cell is assumed as incompressible (${\alpha }~=~1$) so that there are three parameters to be determined in a fitting process. The permeability ($\kappa$) characterizes the flow through the porous elastic meshwork in Darcy’ Law:

1 $$\begin{aligned} v_i =-\kappa p_{,i} \end{aligned}$$

where $v_{i}$ and $p_{,i}$ denote fluid velocity vector component and pore pressure gradient, respectively. The hydraulic permeability $\kappa$ is associated with the intrinsic permeability *k* by:

2 $$\begin{aligned} \kappa =\frac{k}{\eta } \end{aligned}$$

where $\eta$ is the dynamic viscosity of the interstitial liquid assuming it is water in cell, where ${\eta }~=~0.001$ Pas.

Although there is no closed-form analytical solution for a poroelastic half-space indented by a sphere, its numerical solution was developed by Agbezuge and Deresiewicz (1974) and was further explored by Selvadurai (2004) using computational method. If one introduces a dimensionless time parameter $T^{*}$

3 $$\begin{aligned} T^{*}=\sqrt{\frac{2G\kappa t}{R\delta \left( t \right) }} \end{aligned}$$

and a normalized indenter displacement parameter $H^{*}$

4 $$\begin{aligned} H^{*}=\frac{\delta \left( t \right) -\delta \left( 0 \right) }{\delta \left( \infty \right) -\delta \left( 0 \right) } \end{aligned}$$

where *R* is the reduced radius of the tip-cell system ($R~=~[1/R_{1}~+~1/R_{2}]^{-1}$, where $R_{1}$ and $R_{2}$ are the radii of the spherical AFM tip and cell, respectively) and $\delta$(*t*) denotes the time-dependent rigid penetration of the indenter into the cell at a given time *t*. In the present AFM-based indentation on liver cancer cells, the polystyrene indenter is impermeable while the living cell immersed in liquid is considered as permeable. In such a boundary condition and according to the solution by Agbezuge and Deresiewicz (1974), the $H^{*}-T^{*}$ master curve is approximated by a sigmoidal function (Oyen 2008)

5 $$\begin{aligned} H^{*}=A-A\left[ {1+\left( {\frac{T^{*}}{T_0 }} \right) ^{P}} \right] ^{-1} \end{aligned}$$

where *A*, *P* and $T_{0}$ are empirical fitting parameters, given by $A~=~0.928$, $P~=~2.0837$ and $T_{0}~=~0.772$. The shear modulus *G* could be determined from the initial contact radius $a_{0}$ (Selvadurai 2004)

6 $$\begin{aligned} a_0 =\root 3 \of {\frac{3PR}{16G}} \end{aligned}$$

where $a_{0}~=~[{\delta }(0)R]^{1/2}$. The equilibrium Young’s modulus *E* is given by:

7 $$\begin{aligned} E=2\left( {1+\nu } \right) G \end{aligned}$$

The drained Poisson’s ratio of the solid skeleton can be estimated from (Selvadurai 2004)

8 $$\begin{aligned} \delta \left( \infty \right) =\left[ {2\left( {1-\nu } \right) } \right] ^{2/3}\delta \left( 0 \right) \end{aligned}$$

Therefore, combining Eqs. (6) and (8) and fitting the experimental data with Eq. (5) could determine the values for the three key poroelastic parameters *G*, $\nu$ and $\kappa$.

### Statistical analysis

We hypothesize that experimental data fitting the poroelastic model with an $$ R^{2}~>~0.85$$ will be considered as credible (an average $$R^{2}$$ of $$0.90~\pm ~0.05$$ is selected here). Data were evaluated using a paired *t* test to signify statistical differences between two matched data groups, where $$P<~0.05$$ was taken to indicate statistical significance.

**Fig. 3:**
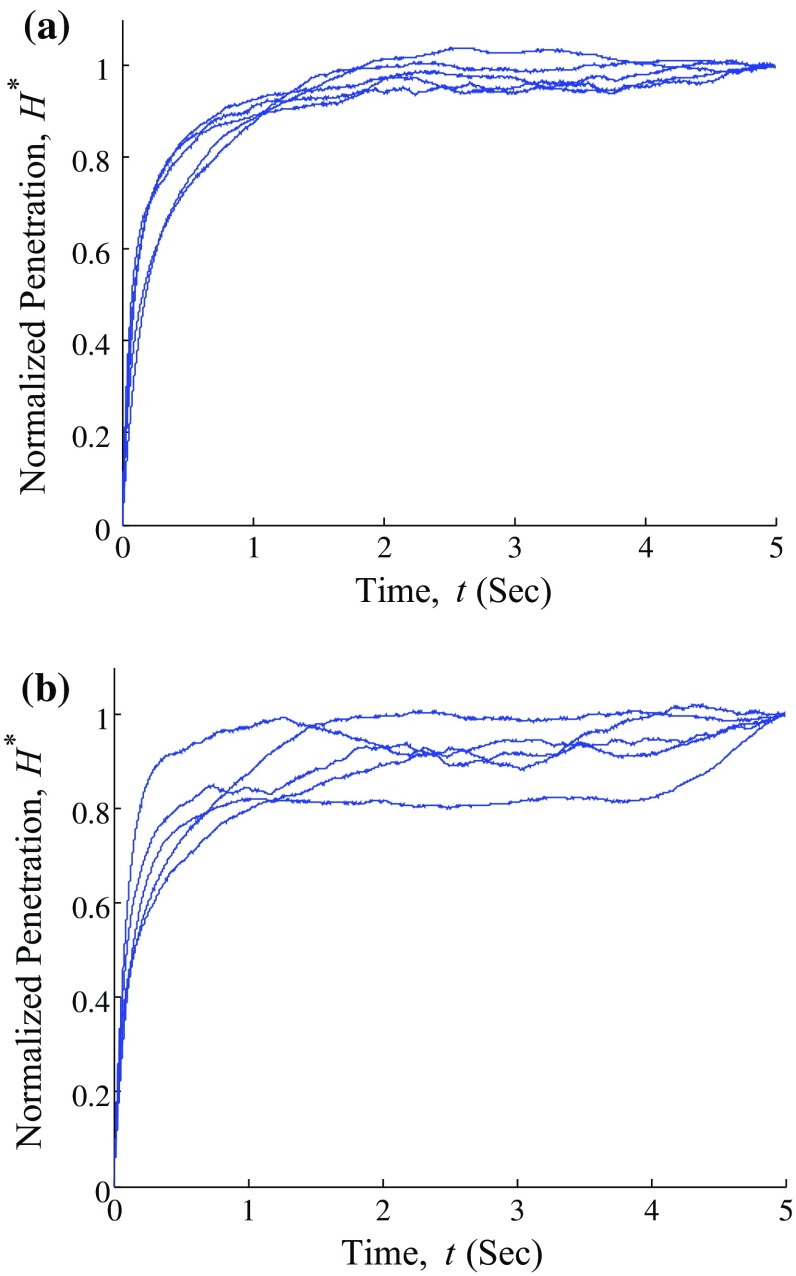
Typical creep curves corresponding to **a** repeated indentations at the same spot and **b** different indentations positions within the same cell

## Results and discussions

### Analysis of the creep curves

In nanoindentation, the measured properties are potentially affected by the substrate effect, which is ignored by following a common rule of thumb that the indentation depth should be less than a tenth of the film thickness (Fischer-Cripps 2000; Cabibil et al. 2001; Jung et al. 2004). In order to investigate the substrate effect, we select an arbitrary intersecting surface, plot the variation of cell height (*H*) and repeated the same creep test illustrated in Fig. 1 along the black line shown in Fig. 2a. $\delta$ denotes the penetration of the indenter into the cell corresponding to equilibrium state, i.e., $\delta$($\infty$). The variation of *H* and $\delta$ on the indentation path is plotted in Fig. 2b. Figure 2c illustrates the variation of $\delta$/*H* along the indentation path, and we can see that $\delta$/*H* exhibits the same increasing trend as $\delta$. In addition, the ratio $\delta$/*H* is below 0.1 over the nucleus region (proximity $10~\upmu \hbox {m}~<~x~<~20~\mu \hbox {m}$) where the extracted poroelastic parameters are in line with each other as shown in Fig. 2d. In addition, both permeability and elastic modulus are apparently even in the nucleus proximity which implies the cell is relatively homogeneous in this area. Therefore, subsequent indentation is performed in this region to avoid substrate effect.

**Fig. 4:**
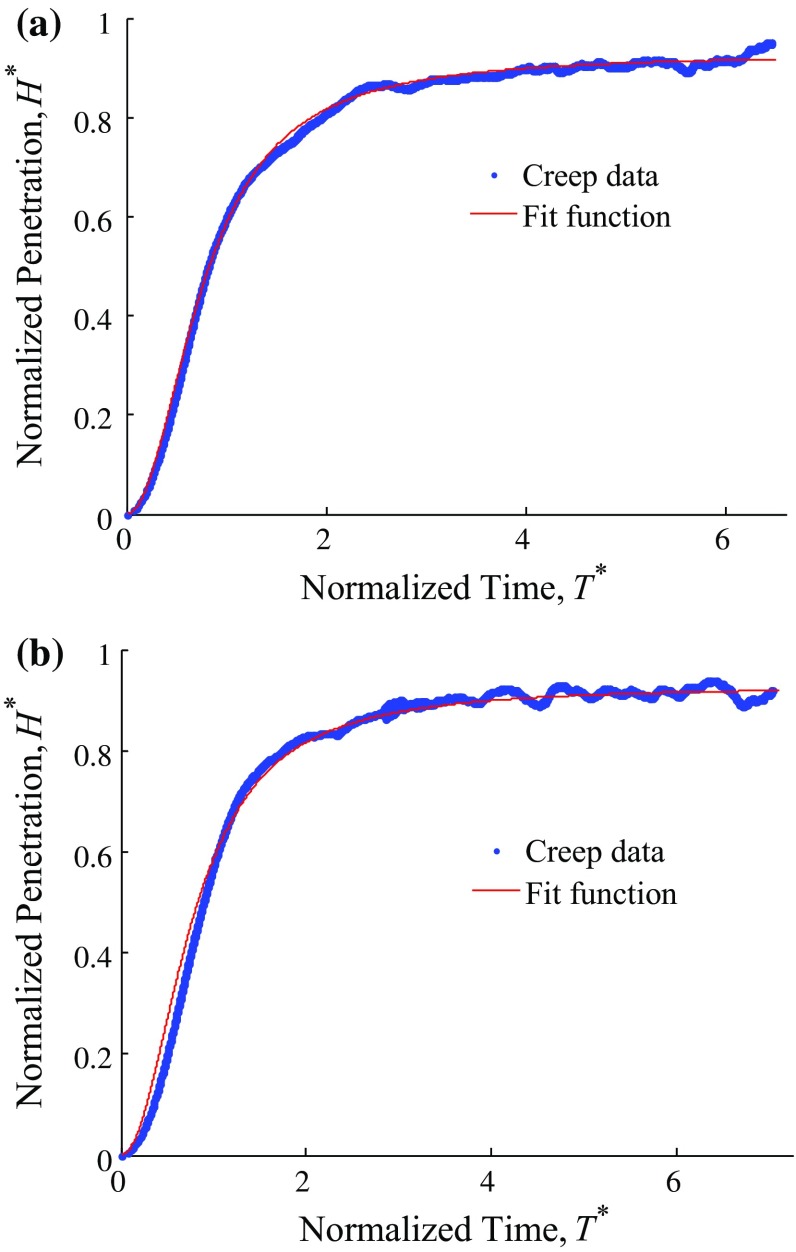
Typical indentation creep curve fitted with the poroelastic model of **a** control and **b** treated cells. The fitting results: **a** control cell, $k~=~3.2\,\times \,10^{-17} \quad m^{2}, D~=~2.56\,\times \,10^{-11} \quad m^{2}/s, G~=~241.3~Pa, {\nu }~=~0.284$, **b** treated cell, $k~=~8.46\,\times \,10^{-17} \quad m^{2}, D~=~10.88\,\times \,10^{-11} \quad m^{2}/s, G~=~127.9~Pa$, ${\nu }~=~0.342$.

In the present AFM-based creep test, we selected five control and treated cells which well attached the bottom of the flask. The indentation position of each cell is done at the cell nucleus proximity for two reasons according to previous analysis. First, this region corresponds to a more thick part of the cell which can alleviate the adverse effect from substrate. Second, the cytoskeleton structure prevailing in this area is more homogeneous and does not exhibit microtubules (Sirghi et al. 2008).

**Fig. 5:**
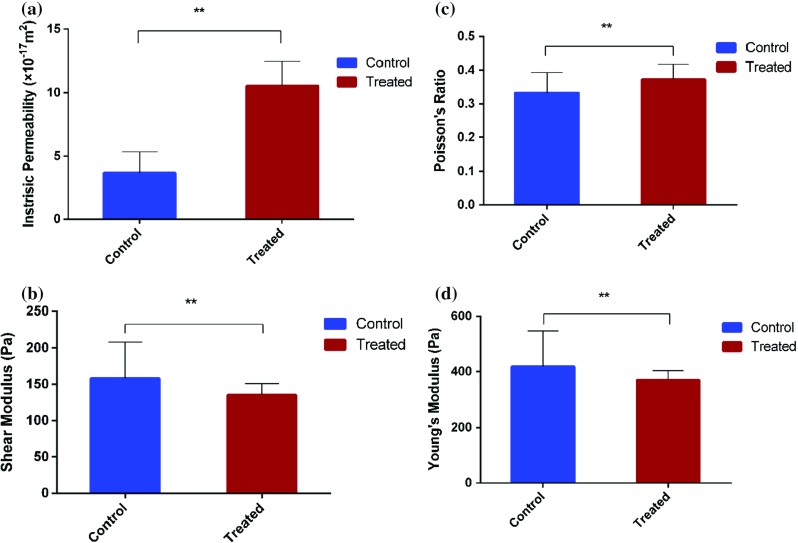
Statistical analysis of **a** intrinsic permeability **b** shear modulus **c** Poisson’s ratio and **d** Young’s modulus of control and treated cells

Each living cell was indented five times at the same location and this was repeated at five different locations over the nucleus region. Since the viability of the SMMC-7721 cells could be affected when exposed in air over a short period of time, there is limited time for us to perform the experiments, and thus a total of 125 indentations were attempted in our investigation. It can be seen the creep curves vary insignificantly in Fig. 3a, in which five creep indentation tests were performed at the same location on the cell. Although Fig.  3a presents the result of indentation at the same location, the time gap between two indentation tests were not controlled accurately. This is likely to introduce an artifact which causes variation between curves shown in Fig. 3a. As the interval between two tests varies, the recovery status of previously deformed cell may not be the same, which is one of the limitation of this study. Figure 3b shows creep curves from five arbitrarily selected locations within the same cell over the nucleus region. It can be seen that indenting on different locations within the same cell, the corresponding creep curves differ only by a small amount to each other. This indicates that the place of the indentation location does not induce significant variation for the concerned measurements. The variation of the $H^{*}$ is up to 0.1 and 0.2 for Fig. 3a and b, respectively, which are likely ascribed to statistical difference and slightly inhomogeneous property in the indented region, respectively. It should be borne in mind that for illustration, Fig. 3 only shows the indentation result of one cell that represents a typical variation of $H^{*}$, and the conclusion obtained above was extended to the other sample cells.

**Fig. 6:**
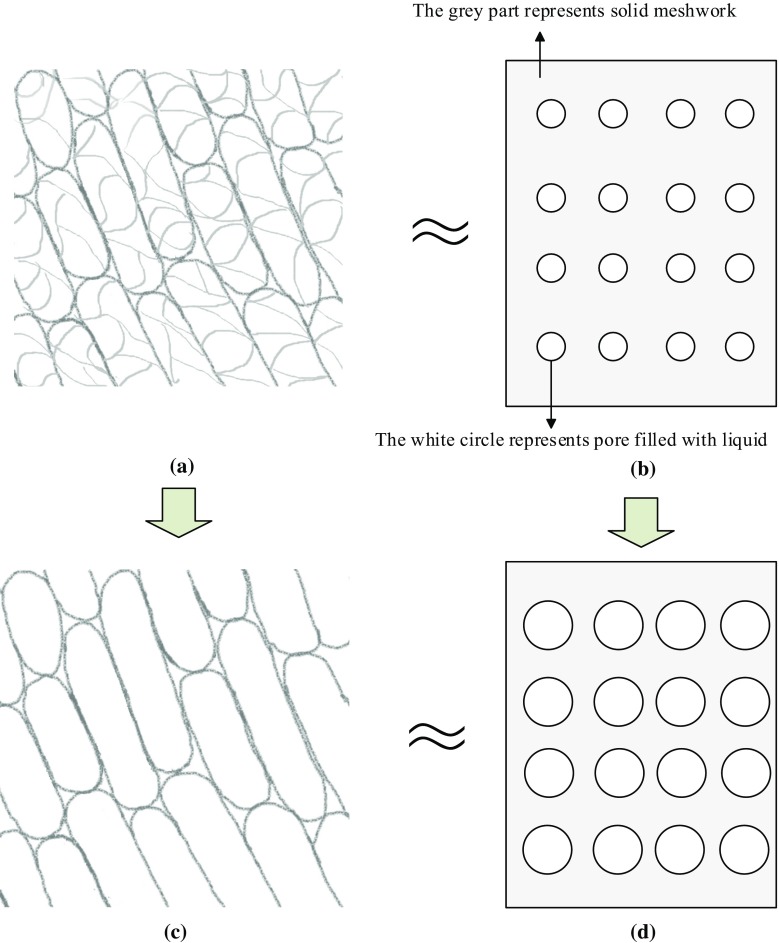
**a** and **b**: the schematic confocal images of filament actin and its equivalent poroelastic material before fullerenol treatment, respectively. **c** and **d**: the schematic confocal images of filament actin and its equivalent poroelastic material after fullerenol treatment, respectively. The filament actin and its proximal interspace are akin to the solid meshwork and pores in the poroelastic material, respectively

A typical normalized displacement-dimensionless time for control and treated cells response (the indentation is performed over the nucleus part of cell) are shown in Fig. 4c and d, respectively, as bold line; the thin line is the function in Eq. (5) used to fit the three key poroelastic parameters *G*, $\nu$ and $\kappa$. At the end of dwell stage ($t~=~5$ s), we assume that the indentation depth has already reached its steady state such that it could be treated as ${\delta }(\infty )$. It can be seen from Fig. 4 that there is a reasonable agreement between the poroelastic model and the shape of the experimental data curves.

### Investigation of cellular poroelastic properties

Figure 5a, b and c illustrate the histograms of the average extracted intrinsic permeability (*k*), shear modulus (*G*) and Poisson’s ratio ($\nu$), respectively. The Young’s modulus is determined by Eq. (7) and its statistical result is illustrated in Fig. 5d. Figure 5a indicates that the permeability of control cell ($3.68\,\times \,10^{-17} \hbox {m}^{2})$ is increased by almost three times to its treated counterpart $(10.55\,\times \,10^{-17}$ $\hbox {m}^{2})$ after fullerenol treatment ($P~<~0.05$). The intrinsic permeability *k* is generally a function of the pore geometry. In particular, it is strongly dependent on porosity $\phi$ which is defined as the ratio of the volume of interconnected pore space to the total porous material. According to the Carman–Kozeny law (Scheidegger 1974) based on the conceptual model of packing of spheres, the intrinsic permeability scales as $k\sim ~{\phi }^{3}/(1 -{\phi })^{2}$. Since higher permeability corresponds to a greater porosity, the statistical results imply that the average size of pores in the cytoskeleton increases after the treatment. On the other hand, the shear modulus, drained Poisson’s ratio and Young’s modulus alter only by a small amount after the treatment, i.e., the first one decreases by 14.45%, the second one increases by 11.75% and the last one decreases by 11.65% as can be seen from Fig. 5b, c and d, respectively  ($P~<~0.05$). This suggests that the fullerenol treatment does not induce significant alternation on the stiffness of the cellular cytoskeleton network. Since the re-arrangement of cytoskeleton could induce alternation of cell elasticity (Keten et al. 2012), it would be rational to conclude that inconspicuous reorganization of cytoskeleton occurs due to the fullerenol treatment.

Although the cellular elasticity and rheology are highly dependent on the cytoskeleton, it is F-actin rather than microtubules nor keratin intermediate filaments that plays the main biological determinant of cellular poroelastic properties (Moeendarbary et al. 2013). The confocal images from the previous report show that almost all the long actin filament bundles collapsed and transformed into punctate structures after the fullerenol treatment (Nie et al. 2017). It resembles the situation that the filament actin partially disappears by comparing schematics shown in Fig. 6a with c, and it is equivalent to the change from Fig. 6b–d where part of the solid meshwork disappears and the pores filled with liquid expands. Therefore, it is rational to observe that the permeability of cell increases significantly while its elastic properties change by a small amount. The experimental results reveal that the changes of actin cytoskeleton treatment with fullerenol resemble the effects of F-actin depolymerization induced by Latrunculin as a common reagent to depolymerize the actin cytoskeleton and affect the poroelastic properties of the cells (Moeendarbary et al. 2013). It is certainly plausible that fullerenol could bind to actin proteins, which potentially impact actin polymerization and depolymerization states (Johnson-Lyles et al. 2010).

Since the present study represents a first attempt to characterize poroelastic properties of liver cancerous cells by using creep test, we cannot compare our results with the counterpart values determined by other authors in similar cell experiments. However, we can compare our results with the values of poroelastic properties of other cells. For example, Berteau et al. (2016) found by stress relaxation the value of intrinsic permeability ranging between $5.5\,\times \,10^{-19} \hbox {m}^{2}$ and $1.59\,\times \,10^{-18} \hbox {m}^{2}$ for mice articular cartilage cells, which almost coincides with the values determined in our study in order of magnitude.

### Verification of cellular poroelastic properties

If a poroelastic material is subjected to a local deformation, it needs time for the interstitial liquid to redistribute. The response of a poroelastic material to deformation depends on the diffusion constant *D*, given by

9 $$\begin{aligned} D=\frac{2\kappa G\left( {1-\nu } \right) \left( {\nu _u -\nu } \right) }{\alpha ^{2}\left( {1-2\nu } \right) ^{2}\left( {1-\nu _u } \right) } \end{aligned}$$

**Fig. 7:**
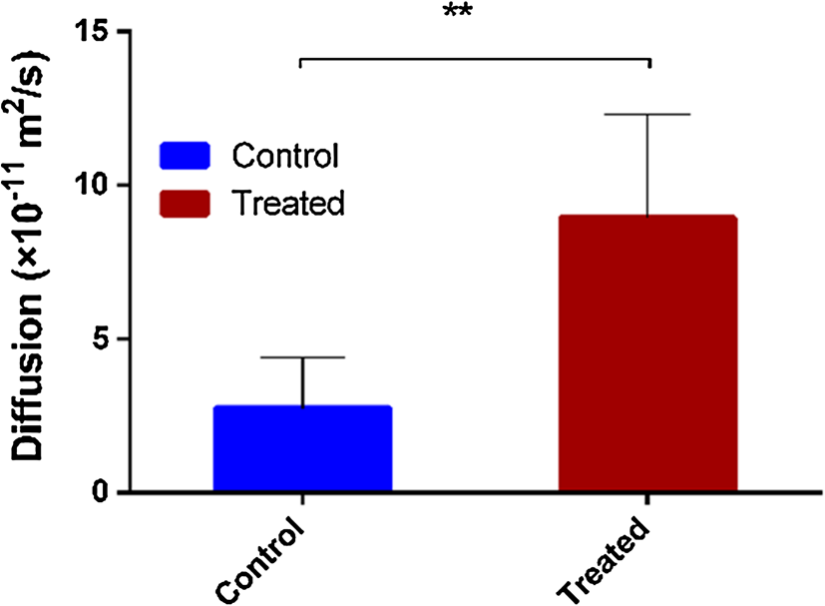
Statistical analysis of average diffusion constant of control and treated cells

This parameter is correlated to the velocity of the consolidation process with larger values corresponding to more rapidly to reach steady-state indentation depth in creep (or stress relaxation). From the poroelastic analysis, one set of value for $\kappa$, *G* and $\nu$ are extracted by fitting each creep curve, and the diffusion constant *D* is derived by Eq. (9). The histogram in Fig. 7 shows that the average diffusion constant of control and treated cells. It can be seen that the treatment increases *k* and $\nu$ and decreases *G*, resulting in an overall increase in *D* by 3.3 times ($P~<~0.05$). In another word, the creep-induced distribution of interstitial fluid takes less time after fullerenol treatment.

The time scale $t_{p}$ for liquid redistribution is given by Moeendarbary et al. (2013)

10 $$\begin{aligned} t_p \approx \frac{L^{2}}{D} \end{aligned}$$

where *L* denotes characteristic length which is assumed to be the one-dimensional projection of the contact area (Heris et al. 2013). According to Hertzian model, *L* is given by

11 $$\begin{aligned} L=2\mathrm{Rarccos}\left[ {\frac{R-\delta \left( \infty \right) }{R}} \right] \end{aligned}$$

where *R* and ${\delta }(\infty )$ denote the radius of spherical indenter and the steady-state indentation depth in creep, respectively. Substituting Eqs. (9) and (11) into Eq. (10) results in a time scale $t_{p}~=~1.7~\pm ~0.2$ s for the control cells and $t_{p}~=~0.8~\pm ~0.3$ s for the treated cells, which are much longer than the ramp time (50 ms). In this regard, the load ramps in a velocity faster than the interstitial cytosol could evacuate out of the porous meshwork, and hence it is fluid redistribution within the cytoplasm that would govern the time-dependent behavior of the cells, i.e., creep in the present study.

### The elastic modulus determined by Hertz contact model

In this section, we performed quasi-static indentation on control and treated cells, and fitted the force (*F*)-penetration ($\delta$) curve by the Hertzian model:

12 $$\begin{aligned} F=\frac{4E}{3\left( {1-\nu ^{2}} \right) }R^{1/2}\delta ^{3/2} \end{aligned}$$

where *E* denotes the Young’s modulus and $\nu$ is Poisson’s ratio which is set to be 0.5 to represent that the living cell is incompressible. Since one of requirements for Hertz contact model is that the contact body should be regarded as homogeneous, the elastic modulus *E* just represents an overall and equivalent value. In the AFM indentation, displacement is controlled and the approaching speed were selected to be 5 and $10~\upmu \hbox {m}/\hbox {s}$.

**Fig. 8:**
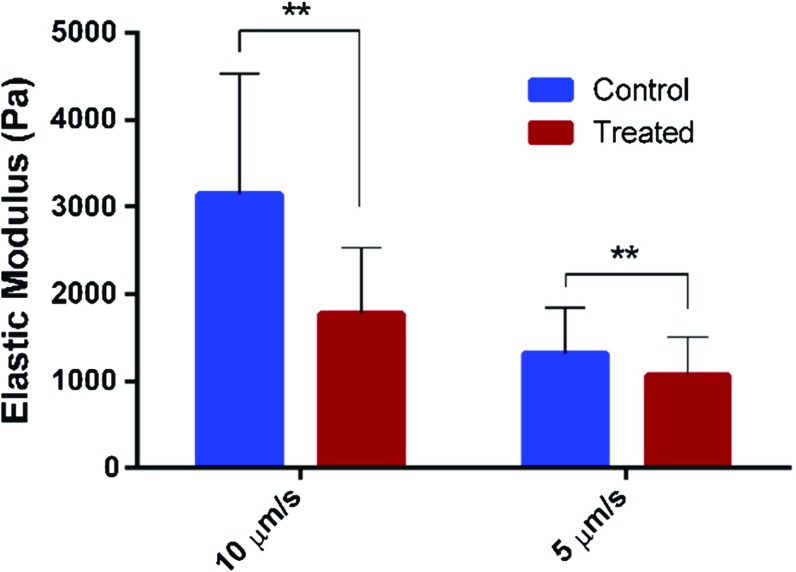
Young’s moduli determined by Hertz model corresponding to different loading rate

Figure 8 shows the statistical results for the average Young’s moduli determined by Hertz model. It can be seen that the Young’s modulus determined by Hertz contact model is loading speed dependent, which is inconsistent with the definition of the elastic modulus as a material’s intrinsic property. In reality, the cytoskeleton is immersed in cytosol which is known as incompressible. Since the probe indents the cell in the speed of several microns per second in Hertz model, the cytosol does not have enough time to escape the pores surrounded by the solid meshwork, i.e., cytoskeleton, and thus they will contribute to the overall cell-stiffness. In this regard, it is more difficult for the liquid to escape the compressed meshwork when the cell is subject to faster loading, which interprets why a higher loading speed would result in a higher effective Young’s modulus as also can be seen in Fig. 8. Although the values for control and treated cell calculated by Hertz model are rate-dependent, the tendency of the modulus between the control and treated cells is the same.

## Conclusion

In this study, AFM-based creep tests were carried out to investigate the poroelastic properties of human hepatocellular carcinoma cell and its fullerenol-treated counterpart. The results show that fullerenol treatment induces a 2.86-fold and 3.26-fold increase in cellular permeability and cytosol diffusion, respectively, while an 11.65% decrease in Young’s modulus. The alternations of these parameters could represent the changes of the cells after fullerenol treatment, and they can also be used to explain cytoskeleton (mainly filaments actin) changes of cells, which is consistent with confocal image results by previous studies. In this sense, the poroelastic model can be used to represent the mechanical property changes of cell’s components of different phases rather than an overall Young’s modulus obtained using an elastic model. This study paves a path of revealing the changes of cytoskeleton due to different drug treatment, cancerization and malignancy, which could provide an instructive method for drug efficacy test, cancer diagnosis and safe therapies.

It is worth noting that further work is required by using different cell lines, drug concentrations and treatment period to confirm its future application. Also there is a limitation in assuming that the biological cells are homogeneous, and therefore an inhomogeneous model considering the surface tension of cyto-membrane will be developed in our future studies.

## References

[CR1] Agbezuge LK, Deresiewicz H (1974). On the indentation of a consolidating half-space. Israel J Technol.

[CR2] Aryaei A, Jayasuriya AC (2013). Mechanical properties of human amniotic fluid stem cells using nanoindentation. J Biomech.

[CR3] Berteau JP, Oyen M, Shefelbine SJ (2016). Permeability and shear modulus of articular cartilage in growing mice. Biomech Model Mechan.

[CR4] Bosi S, Da Ros T, Spalluto G (2003). Fullerene derivatives: an attractive tool for biological applications. Eur J Med Chem.

[CR5] Cabibil H, Celio H, Lozano J, White JM, Winter RM (2001). Nanomechanical properties of polysiloxane-oxide interphases measured by interfacial force microscopy. Langmuir.

[CR6] Chen J (2014). Nanobiomechanics of living cells: a review. J R Soc Interface.

[CR7] Chen Z, Ma L, Liu Y, Chen C (2012). Applications of functionalized fullerenes in tumor theranostics. Theranostics.

[CR8] Etienne-Manneville S (2004). Actin and microtubules in cell motility: which one is in control?. Traffic.

[CR9] Fischer-Cripps AC (2000). A review of analysis methods for sub-micro indentation testing. Vacuum.

[CR10] Hawkins T, Mirigian M, Yasar MS, Ross JL (2010). Mechanics of microtubules. J Biomech.

[CR11] Heris HK, Miri AK, Tripathy U, Barthelat F, Mongeau L (2013). Indentation of poroviscoelastic vocal fold tissue using an atomic force microscope. J Mech Behav Biomed Mater.

[CR12] Johnson-Lyles DN, Peifley K, Lockett S, Neun BW, Hansen M, Clogston J, Stern ST, McNeil SE (2010). Fullerenol Cytotoxicity in kidney cells is associated with cytoskeleton disruption, autophagic vacuole accumulation, and mitochondrial dysfunction. Toxicol Appl Pharmacol.

[CR13] Jung YG, Lawn BR, Martyniuk M, Huang H, Hu XZ (2004). Evaluation of elastic modulus and hardness of thin films by nanoindentation. J Mater Res.

[CR14] Ketene AN, Roberts PC, Shea AA, Schmelz EM, Agah M (2012) Agah: Actin filaments play a primary role for structural integrity and viscoelastic response in cells. Integr Biol (Camb) 4:540–549 10.1039/c2ib00168c 22446682

[CR15] Leipzig ND, Athanasiou KA (2005). Unconfined creep compression of chondrocytes. J Biomech.

[CR16] Lu LH, Lee YT, Chen HW, Long YC, Huang HC (1998). The possible mechanisms of the antiproliferative effect of fullerenol, polyhydroxylated C$$_{60}$$, on vascular smooth muscle cells. Brit J Pharmacol.

[CR17] Moeendarbary E, Valon L, Fritzsche M, Harris AR, Moulding DA, Thrasher AJ, Stride E, Mahadevan L, Charras GT (2013). The cytoplasm of living cells behaves as a poroelastic material. Nat Mater.

[CR18] Mrdanović J, Solajić S, Bogdanović V, Stankov K, Bogdanovicć G, Djordjevic A (2009). Effects of fullerenol $$\text{C}_{60}$$(OH)$$_{24}$$ on the frequency of micronuclei and chromosome aberrations in CHO-K1 cells. Mutat Res-Gen Tox En.

[CR19] Neumann T (2008) JPK Instruments Application Report

[CR20] Nie X, Tang JL, Liu Y, Cai R, Miao Q, Zhao YL, CHen CY (2017) Fullerenol inhibits the cross-talk between bone marrow-derived mesenchymal stem cells and tumor cells by regulating MAPK signaling. Nanomed-Nanotechnol 13:1879–1890 10.1016/j.nano.2017.03.013 28365417

[CR21] Nikolaev NI, Müller T, Williams DJ, Liu Y (2014). Changes in the stiffness of human mesenchymal stem cells with the progress of cell death as measured by atomic force microscopy. J Biomech.

[CR22] Oyen M (2008). Poroelastic nanoindentation responses of hydrated bone. J Mater Res.

[CR23] Partha R, Conyers JL (2009). Biomedical applications of functionalized fullerene-based nanomaterials. Int J Nanomed.

[CR24] Rade I, Biljana G, Aleksandar D, Borut S (2008). Bioapplication and activity of fullerenol $$\text{ C }_{60}$$(OH)$$_{24}$$. Afr J Biotechnol.

[CR25] Scheidegger AE (1974). The physics of flow through porous media.

[CR26] Sirghi L, Ponti J, Broggi F, Rossi F (2008). Probing elasticity and adhesion of live cells by atomic force microscopy indentation. Eur Biophys J.

[CR27] Selvadurai APS (2004). Stationary damage modeling of poroelastic contact. Int J Solids Struct.

[CR28] Unterberger MJ, Schmoller KM, Bausch AR, Holzapfel GA (2013) A new approach to model cross-linked actin networks: multi-scale continuum formulation and computational analysis. J Mech Behav Biomed 22:95–114 10.1016/j.jmbbm.2012.11.019 23601624

[CR29] Wang HF (2000). Theory of linear poroelasticity with applications to geomechanics and hydrogeology.

